# Understanding and Modeling Metastasis Biology to Improve Therapeutic Strategies for Combating Osteosarcoma Progression

**DOI:** 10.3389/fonc.2020.00013

**Published:** 2020-01-31

**Authors:** Timothy M. Fan, Ryan D. Roberts, Michael M. Lizardo

**Affiliations:** ^1^Comparative Oncology Research Laboratory, Department of Veterinary Clinical Medicine, College of Veterinary Medicine, University of Illinois at Urbana-Champaign, Urbana, IL, United States; ^2^Center for Childhood Cancer and Blood Disorders, Abigail Wexner Research Institute at Nationwide Children's Hospital, The James Comprehensive Cancer Center at The Ohio State University, Columbus, OH, United States; ^3^Poul Sorensen Laboratory, Department of Molecular Oncology, BC Cancer, Part of the Provincial Health Services Authority in British Columbia, Vancouver, BC, Canada

**Keywords:** comparative oncology, metastasis biology, experimental models, translational therapeutics, canine cancer

## Abstract

Osteosarcoma is a malignant primary tumor of bone, arising from transformed progenitor cells with osteoblastic differentiation and osteoid production. While categorized as a rare tumor, most patients diagnosed with osteosarcoma are adolescents in their second decade of life and underscores the potential for life changing consequences in this vulnerable population. In the setting of localized disease, conventional treatment for osteosarcoma affords a cure rate approaching 70%; however, survival for patients suffering from metastatic disease remain disappointing with only 20% of individuals being alive past 5 years post-diagnosis. In patients with incurable disease, pulmonary metastases remain the leading cause for osteosarcoma-associated mortality; yet identifying new strategies for combating metastatic progression remains at a scientific and clinical impasse, with no significant advancements for the past four decades. While there is resonating clinical urgency for newer and more effective treatment options for managing osteosarcoma metastases, the discovery of druggable targets and development of innovative therapies for inhibiting metastatic progression will require a deeper and more detailed understanding of osteosarcoma metastasis biology. Toward the goal of illuminating the processes involved in cancer metastasis, a convergent science approach inclusive of diverse disciplines spanning the biology and physical science domains can offer novel and synergistic perspectives, inventive, and sophisticated model systems, and disruptive experimental approaches that can accelerate the discovery and characterization of key processes operative during metastatic progression. Through the lens of trans-disciplinary research, the field of comparative oncology is uniquely positioned to advance new discoveries in metastasis biology toward impactful clinical translation through the inclusion of pet dogs diagnosed with metastatic osteosarcoma. Given the spontaneous course of osteosarcoma development in the context of real-time tumor microenvironmental cues and immune mechanisms, pet dogs are distinctively valuable in translational modeling given their faithful recapitulation of metastatic disease progression as occurs in humans. Pet dogs can be leveraged for the exploration of novel therapies that exploit tumor cell vulnerabilities, perturb local microenvironmental cues, and amplify immunologic recognition. In this capacity, pet dogs can serve as valuable corroborative models for realizing the science and best clinical practices necessary for understanding and combating osteosarcoma metastases.

## Targeting Pulmonary Metastasis in Osteosarcoma

Since the institution of chemotherapy in the 1960s, relapse-free survival for osteosarcoma (OS) patients with localized disease has dramatically improved. The current standard of care involves surgical resection of the primary tumor and multi-agent chemotherapy (both in the neoadjuvant and adjuvant setting) which can result in 5-year survival rates up to 70% for patients with localized disease (1). For those patients who present with distant metastases (usually in the lung), outcomes are much poorer with a survival rate of about 20% (2). The negative prognoses associated with macroscopic disseminated OS burdens is not unique, but rather holds true for many types of cancers that metastasize (3); and underscores the broader need in combating metastatic progression across diverse solid tumor histologies. For OS patients, major hurdles that reduce overall survival include relapse, which occurs in 1/3 of patients with localized disease (4) and in the majority (~75%) of patients presenting with systemic disease (5); and the development of chemo-resistance (6). Since overall survival rates have plateaued with multi-agent chemotherapy (7), there remains an impetus to discover and clinically deploy alternative non-cytotoxin based anti-metastatic therapeutics that inhibit lung metastasis progression and may lead to improved patient outcomes. Several investigators in the metastasis research community have advocated the idea that delaying or inhibiting metastatic progression (particularly the early stages of lung colonization) should be the most clinical and biologic relevant metric rather than the cytoreduction of the primary tumor in the evaluation of new drugs (3, 8, 9). The merit of this proposed paradigm shift in therapeutic assessment is supported by historical clinical data that micrometastases in the lung are already present in OS patients with localized tumors and that adjuvant chemotherapy has been shown to improve relapse-free survival (10). Additionally, preclinical effectiveness of molecularly-targeted therapy for targeting early stages of lung colonization or micrometastases have been shown previously (11, 12) (also see **Table 2**), and justify the exploration of precision medicine approaches for improving survival outcomes. To accelerate discovery to impact, the rational development of anti-metastatic therapeutics requires a convergent science approach including (1) a better understanding of OS metastasis biology in relation to the lung microenvironment and (2) the availability of engineered and natural model systems that most faithfully recapitulate the complexities of metastatic progression. Through transdisciplinary collaborative research, it is envisioned that novel and effective anti-metastatic therapeutics can be identified and translated to extend the lives of patients with OS by eradicating or thwarting the progression of subclinical micrometastatic disease that persisted following standard multi-agent chemotherapy.

## Biology of Pulmonary Metastases

### Dissemination From the Primary Tumor

**Figure 1:**
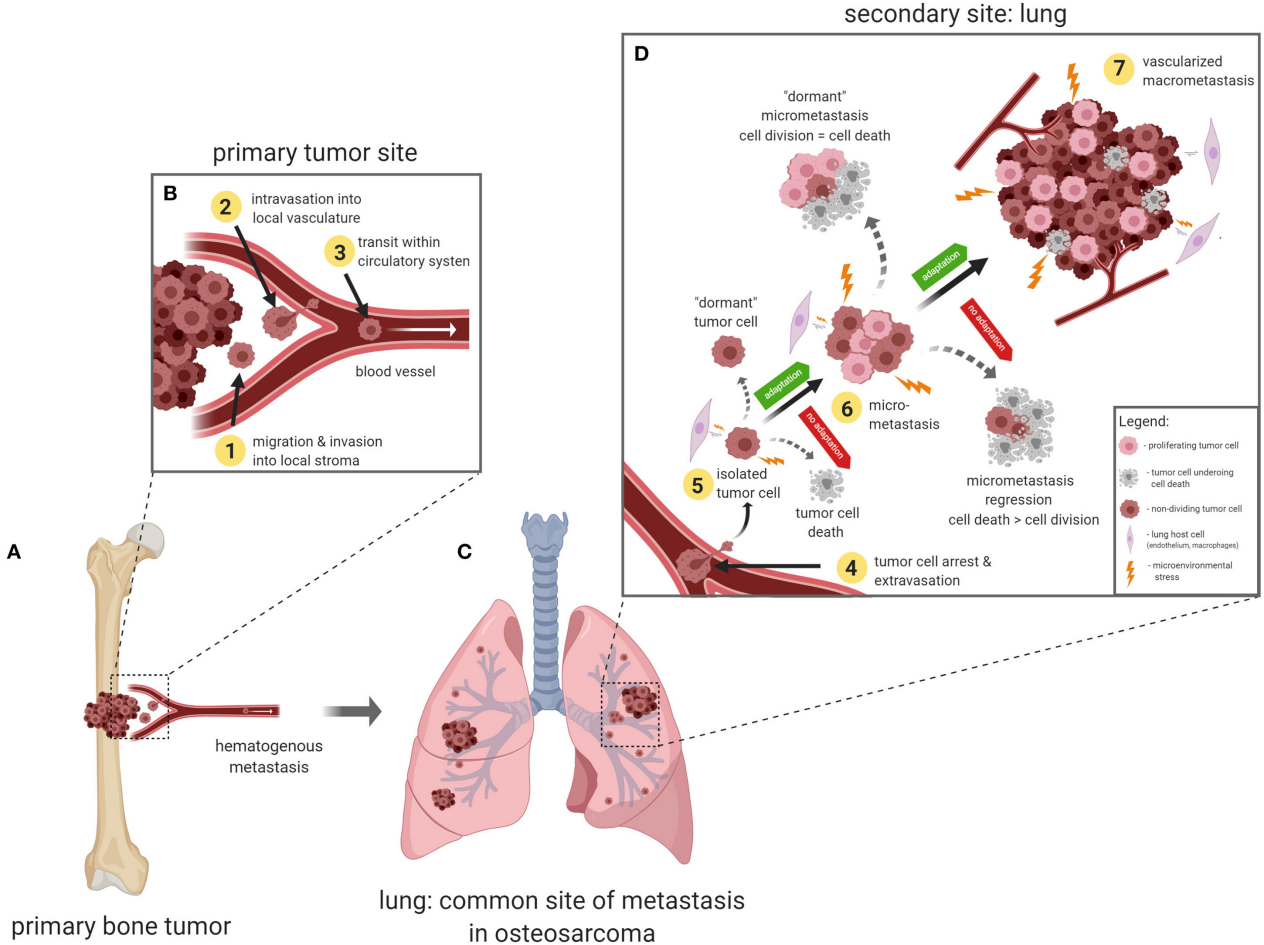
The metastatic cascade in osteosarcoma. **(A)** Primary OS tumor, usually in the long bones. **(B)** Tumor cells acquire an invasive phenotype and migrate away from the primary tumor and invade into surrounding tissues (step 1). Tumor cells interact with the basement membrane and endothelial cells to intravasate into the blood microvasculature (step 2) and travel in the circulation (step 3). **(C)** Upon arrival at the secondary site (lung), tumor cells arrest via size restriction or adhesion interactions with the pulmonary microvascular endothelial cells (step 4). **(D)** Once tumor cells extravasate out of the blood vessels, they must be able to adapt and survive in the lung microenvironment (step 5). At this vulnerable stage, tumor cells can undergo a number of fates which include- enter cellular dormancy, die off, or if the stresses of the lung microenvironment can be successfully managed, tumor cells can proliferate into multi-cellular micrometastases (step 6). Micrometastases can enter into a state of “angiogenic dormancy” and remain the same size, or regress if cell death is greater than proliferation, or recruit local blood vessels and form a vascularized secondary tumor (step 7).

The metastatic process, or more commonly referred to as the *metastatic cascade*, describes the progressive steps of tumor cell dissemination from the primary tumor, transit within the blood vasculature, and the establishment of clinically detectable pulmonary metastases (Figure 1). Since each step of the metastatic cascade is rate limiting, metastasis is considered to be a very inefficient process (30–32). The initial stages of metastasis involve the acquisition of an invasive phenotype and migration away from the primary tumor site (step 1, Figures 1A,B). Several studies have shown that OS cells secrete proteolytic enzymes such as matrix metalloproteinases (MMPs) and cathepsins which causes the degradation of local tissue extracellular matrix (ECM) and basement membranes (33). Modulation of TIMP3, MMP1, MMP3, MMP11 have been shown to influence *in vitro* invasiveness of OS cells, and enhance *in vivo* tumorigenicity (34–36). OS cell interactions with local stromal cells such as mesenchymal stem cells (37) and endothelial cells (38, 39), have been found to be pro-tumorigenic, whereas interactions with natural killer cells (40) or primed dendritic cells (41), were shown to have anti-tumor effects.

### Intravasation and Transit Within the Blood Vasculature

Once tumor cells reach the local microvasculature, intravasation, or entry into blood vessels, is the next step in the metastatic cascade (step 2, Figures 1A,B). Entry into the local microvasculature requires OS cell interaction with endothelial cells. Several *in vitro* models exist to study tumor cell interactions with endothelial cells (42), with the simplest system being the co-culturing of tumor cells onto a monolayer of endothelial cells. Research from several groups have utilized this *in vitro* co-culture method and have shown that RUNX and osteopontin (43), uPAR (14), and α_v_β_3_ (44) influence the physical interactions between OS cells and endothelial cells. More importantly, several of these studies have shown that interfering with these OS cell-endothelial interactions were found to inhibit metastasis formation *in vivo* (14, 43).

Once within the blood stream, OS cells must be able to resist *anoikis*, a specialized form of apoptosis induced by the disruption of cell-matrix interactions, as first described by Frisch and Francis (45). A number of key regulators of anoikis have been characterized since its initial discovery (e.g., Mcl-1, Cav-1, Bcl-x_L_, c-FLIP) (46) and several of these genes have been linked to metastatic capacity in breast cancer cells (47) and OS cells (24, 48, 49).

Another type of stress OS cells encounter within the circulation is the physical hemodynamic forces of blood flow. Observations on the hemodynamic destruction of tumor cells were initially made by Weiss and Dimitrov (50). The major physical stressor in the blood circulation is fluid shear stress (FSS), which is defined as the frictional forces between moving layers, and is measured in Newtons per meter squared (N/m^2^) or Dynes per centimeter squared (Dyn/cm^2^) (51). FSS in the blood circulation ranges from 1 to 30 Dyn/cm^2^ depending on the anatomical location (52). Lien and colleagues have demonstrated that OS cells (MG63) were found to have higher levels of apoptosis when exposed to FSS ranging from 0.5 to 12 Dyn/cm^2^ when compared to control static cells in an *in vitro* flow chamber (53). The authors also demonstrated that the level of OS apoptosis correlated with increasing times of exposure of various FSS conditions. It would be interesting to assess whether MG63.3 cells, a highly metastatic variant of MG63 cells, characterized by Ren et al. (54), exhibit some level of resistance to FSS-induced apoptosis.

### Lung Colonization and Microenvironmental Stressors

If OS cells can resist anoikis and adapt to damaging FSS in the blood circulation, arrest, and survival in the lung microvasculature presents the next significant challenge to metastatic OS cells. Several studies using the experimental metastasis model (tail vein injection of tumor cells) have demonstrated that the majority of tumor cells that arrive in the lung do not survive, and only a small subset of the initial population (1–6%) were able to successfully establish metastases (31, 32). These studies have carefully analyzed tumor cell fate over time and concluded that metastatic colonization of the lung is a non-linear process where tumor cells can undergo any number of fates, as illustrated in Figure 1D. Newly arrested tumor cells can either: (1) enter a dormant, viable but non-dividing state, as observed in several lung metastasis studies (32, 55, 56); (2) proliferate into a pre-angiogenic micrometastasis, or (3) undergo apoptotic cell death (57, 58). Micrometastases, in turn, can also undergo a number of fates which include: (1) enter a state “angiogenic dormancy” where tumor cell proliferation is balanced with cell death (59), (2) proliferate into a vascularized macrometastatic lesion (60), or (3) regress if tumor cell death is greater than cell proliferation. The ability to adapt quickly to a harsh new microenvironment is a prerequisite for metastatic cancer cell survival and proliferation in the lung. Stress adaptation pathways depend on the nature of the particular stress encountered, and several research groups have begun to shed light on this aspect of metastasis biology.

Redox stress is a major microenvironmental stressor that contributes to tumor cell clearance in the lung since several studies have provided microscopic imaging evidence and “omic” data supporting this notion. Qiu et al. (58) have shown that the physical arrest of murine melanoma cells in the lung stimulates the local microvascular endothelial cells to release a burst of nitric oxide (NO), which was cytotoxic to tumor cells. Inhibition of NO release by L-NAME (a nitric oxide synthase inhibitor) treatment or the use of endothelial nitric oxide synthase knock-out mice resulted in higher lung tumor burden. Piskounova et al. (61) have shown that metastatic melanoma cells adapt to the redox stress in the lung by upregulating the NADPH-generating enzyme ALDH1L2; and targeting shRNAs against ALDH1L2 resulted in lower lung tumor burden (61). NADPH is important in maintaining redox homeostasis (62), and a recent study by Basnet et al. (63) have shown that micrometastases of breast cancer cells in the mouse lung have elevated transcript and protein levels of antioxidant genes (e.g., NRF2 and GPX1). The notion that ROS can negatively regulate metastasis formation is somewhat controversial since other studies seem to suggest the opposite (64–66). These discrepancies may be due to cell type-specific responses, or the particular dose of ROS exposure. Low, sublethal concentrations of ROS can turn on antioxidant responses, whereas high concentrations of ROS can cause irreversible damage to proteins, lipids and DNA with consequent cell death.

Another type of cellular stress closely linked to redox stress is endoplasmic reticulum (ER) stress. Protein folding processes within the ER are exquisitely sensitive to perturbations in cellular redox state, Ca^2+^ concentration within ER lumen, and ATP supply (67). Redox stress can alter the oxidative protein folding environment of the ER lumen, which results in the accumulation of unfolded proteins, a condition known as ER stress (68). The unfolded protein response (UPR) is activated by various sensors on the ER membrane, and an adaptive transcriptional program is activated to increase the chaperone capacity of the ER and increase ER-associated degradation pathways to compensate for the sudden load of unfolded proteins (67). The UPR has been found to be dysregulated in many types of cancer, including OS (69, 70). Several highly metastatic human OS cell lines were found to upregulate the ER chaperone protein GRP78 at higher levels compared to their low metastatic counterparts during ER stress (12), and shRNAs and IT-139, a small molecule inhibitor of GRP78 under clinical investigation (71), were found to reduce lung metastatic burden. Translocation of the transcription factor ATF6α to the nucleus is also part of ER stress response, and human OS cells were found to have elevated levels of nuclear ATF6α compared to osteoblast controls under ER stress conditions (72). Downstream targets of ATF6α such as GRP78, PDI, and ERO1β where found to confer chemotherapy resistance in OS cells, and down-modulation of ATF6α resulted in increased sensitivity to cisplatin. Moreover, elevated levels of ATF6α in patient samples was predictive or poorer overall survival and poorer response to chemotherapy (72). Several groups have also found UPR-related pathways to be dysregulated in OS (73–75).

Although the microenvironmental stressors discussed above can contribute to tumor cell clearance in the lung, these observations do not explain the apparent “organotropism” of OS cells for the lung. Why do metastases in OS preferentially occur in the lung? The answer, in part, may be due to mechanical restriction of disseminated tumor cells in the lung microvasculature. Human alveolar capillaries range from 5 to 8 μm in diameter (76), whereas the average diameter of osteoblastic osteosarcoma cells ranges from 10 to 19 μm (estimated from histology micrographs) (77). Circulating tumor cells often arrest via size restriction within the first microvascular capillary bed they encounter, and video microscopic evidence from animal studies suggest that organs such as the lung and liver are efficient at “filtering” out circulating tumor cells from the blood (78). OS cell “organotropism” for the lung can also be explained by the concept of the “pre-metastatic niche” (PMN), in which growth factors from the primary tumor “prime” downstream metastatic sites for tumor cell engraftment (79–81). As to whether PMN contributes to lung colonization in OS, Murgai et al. (82) found that metastatic OS cells secret exosomes containing cytokines that can induce lung perivascular cells to secrete fibronectin. The same authors also demonstrated that fibronectin promoted tumor cell adhesion, migration, and proliferation *in vitro*. Additionally, Macklin et al. (83) also found that highly metastatic OS cells are capable of secreting extracellular vesicles that were preferentially retained in the lung, but not liver. More definitive studies will be needed to define OS-specific changes in the lung during PMN formation, and whether or not modulation of OS-specific PMN can influence the formation of lung metastasis.

## Model Systems to Study Osteosarcoma Metastases

### Preclinical Models to Study and Image the Steps of Metastasis

Since lung metastasis progression involves complex 3 dimensional (3D) interactions between OS cells, ECM, and lung parenchymal cells, model systems that can maintain or partially recapitulate some aspects of these 3D interactions will allow researchers to interpret metastatic OS cell responses to gene therapy or pharmacologic intervention in a relevant microenvironmental context. Indeed, several studies have demonstrated that tumor cell response to therapeutics differ when comparing 2D vs. 3D growth conditions (84–86). To this end, several microscope-based models exist that permit researchers to directly visualize and study metastatic cancer cell behavior in a 3D microenvironment. Such models are described below, and the benefits and limitations of each model are discussed.

#### Chick Chorioallantoic Membrane Model

**Figure 2:**
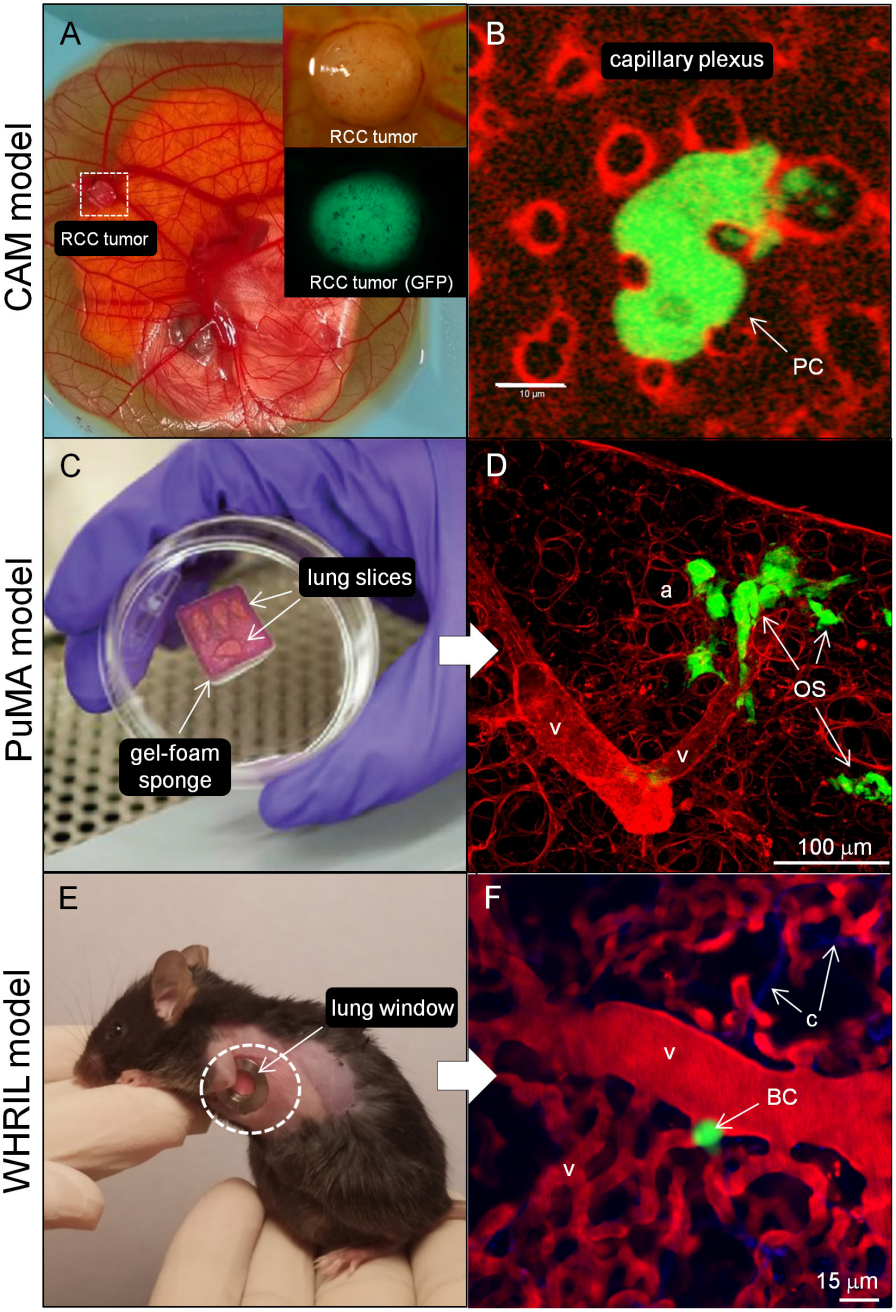
Imaging models to study the metastatic cascade in cancer. **(A)** The CAM model (whole mount image) showing the chick embryo and highly vascularized CAM. A small renal cell carcinoma (RCC) explant can be seen growing in the dashed white box. Zoomed image of a different established GFP-expressing RCC tumor where the entire tumor, associated vasculature, and corresponding fluorescence image (below) are shown (Image courtesy of Matthew Lowerison and Pengfei Song, UIUC). **(B)** High magnification, single cell imaging of a GFP-labeled prostatic carcinoma cell (PC) migrating through capillary plexus (labeled with rhodamine-lectin) and forming invadopodia (yellow arrowheads) into the lumen of 2 adjacent capillaries in the CAM model (Image courtesy of Fabrice Lucien and Yohan Kim, Mayo Clinic). **(C)** The PuMA is an *ex vivo* lung explant model where tumor cells in viable lung tissue is maintained in cell culture. The lung slices are kept at an air-liquid interface on top of a gelatin sponge. **(D)** Shows the lung parenchyma (stained red with DAR4M) and eGFP-expressing MG63 OS cells (OS) interacting with vessel-like structures (v). Scalebar = 100 μm. See Lizardo and Sorensen (88) for methods. **(E)** The WHRIL model allows for the direct visualize of lung tissue through a window in the mouse chest cavity (89) as shown with the dashed white circle. **(F)** Fluorescent micrograph showing the lung microvasculature (v) (labeled red with tetramethylrhodamine) and GFP-expressing breast cancer cell (BC). Blue fibers represent second harmonic imaging of connective tissue (c) fibers. Scalebar = 15 μm (Images courtesy of David R. Entenberg, Albert Einstein College of Medicine). UIUC, University of Illinois at Urbana-Champaign.

The chick chorioallantoic membrane (CAM) is a highly vascularized membrane that primarily functions as a gas-exchange organ for the developing embryo (87). The CAM is commonly studied in a shell-less format (*ex ovo*), where xenograft human tumor fragments or a tumor cell suspension (Figure 2A) can be placed onto the CAM or injected into blood vessels of the CAM. The CAM has proven to be a useful model in studying tumor angiogenesis (90, 91), tumor cell migration and invasion (92), intravasation into blood vessels (93), tumor cells in transit within the vasculature, extravasation out from blood vessels (Figure 2B) (94–98), and the outgrowth of patient derived xenografts (99). In OS research, the CAM model has been used to study tumor growth of a variety of OS cell lines (100), angiogenesis (101), and metastasis to distant sites (102). The main benefits of using the chick CAM model include: (1) amenable to *in vivo* imaging, (2) relatively inexpensive, and (3) can be used for high-throughput screening of targeted therapies. Disadvantages of the CAM model include: (1) short observation times (days), (2) inability to study tumor cell interactions with the immune system since the chick CAM is immunodeficient until developmental day 18 (87), and (3) fewer antibodies available for host chicken antigens (103). The chick CAM model is applicable to the study of tumor cell invasion (step 1, Figure 1B), interactions between endothelial cells during intravasation, transit within blood vessels, and extravasation (steps 2, 3, and 4, Figures 1B,D) since these steps are readily observable at the surface of the chick CAM. Tumor colonization of distant sites in the chick CAM are not accessible for imaging, and thus harvesting the organs for histology, or polymerase chain reaction assays for tumor specific DNA sequences are required. To study lung colonization in OS, other microscope-based models that can examine lung tissue would be more appropriate.

#### Pulmonary Metastasis Assay (PuMA)

A technical advance that addresses the need to directly visualize and characterize the growth of metastatic OS cells in the lung microenvironment is called the pulmonary metastasis assay (PuMA), first developed by Mendoza et al. (104), and further refined by others (88, 105). The PuMA is an *ex vivo*, lung tissue explant model where fluorescently labeled tumor cells in viable lung tissue (Figures 2C,D) can be maintained *in vitro* for up to 21 days of observation. High and low metastatic pairs of human and mouse OS cell lines, whose *in vivo* metastatic phenotypes were characterized elsewhere (54), retain their metastatic propensities in the *ex vivo* PuMA model. Such observations suggest that despite the lack of blood flow, certain cellular, and extracellular features of lung tissue still exert “microenvironmental pressures” that are not conducive to the growth of low metastatic tumor cells, but still permit the growth of highly metastatic tumor cells. Indeed, the histology and microarchitecture of PuMA tissue sections are virtually indistinguishable from that of *in vivo* lungs (104). The PuMA model has been used to assess how gene modulation or drug treatment affects metastatic OS growth in lung tissue (12, 21–23, 25, 106). The PuMA model has several advantages which include: (1) the ability to directly study metastatic OS cells at both the cellular and subcellular level while in a relevant 3D microenvironment, (2) amenable to molecular imaging (gene or signaling pathways) by labeling tumor cells with fluorescent dyes, fluorescent protein reporter or protein fusion constructs, and (3) the PuMA model has recently been adapted to a 96-well plate format for a high-throughput drug screen (20). For image analysis, proprietary software is not needed, and analysis can be done with publicly available software packages such as ImageJ. Image processing can be expedited through automation as described by Young et al. (105). One major drawback of the PuMA model is the limited number of cell lines that are compatible with the assay. For tumor cell lines that have not been previously published to work within the PuMA model, researchers must empirically determine whether their metastatic tumor cell line of interest is compatible with the B-media used in the PuMA model, and whether their cell line can grow into progressively larger lesions over time. Secondly, the length of observation in the PuMA model is limited to 21 days post-injection of tumor cells. Beyond 21 days, the PuMA lung tissue becomes devoid of lung parenchymal cells, leaving only connective tissue. The PuMA model is ideal in studying tumor cell arrest in the lung microvasculature, extravasation, interactions with the lung parenchyma, and the formation of micrometastases (steps 4, 5, and 6, Figure 1D). If a researcher's investigations require an intact microcirculation, then an intravital (within a living subject) imaging model of the lung would be more appropriate.

#### Intravital Video Microscopy of Lung Metastasis

Direct observation of labeled tumor cells in an intact lung perfusion model have been described previously (58, 107); however this method is an *ex vivo* perfusion model where the lungs were removed *en bloc* and imaged on an inverted microscope. Intravital imaging of the microcirculation of various organs (such as lung or liver) was reported by Varghese et al. (108), where an acute preparation of the organ of interest was stably imaged on an inverted microscope for 4–6 h in anesthetized mice. While innovative at the time, this technique was prone to motion artifact from physiologic processes such as breathing or heart beating, and necessitated movement artifact compensation through a post-processing image stabilization algorithm. More recently, imaging of labeled tumor cells in the lung of a live, free breathing mice was recently described by Entenberg et al. (89). In this intravital video microscopy model, called Window for High-Resolution Imaging in the Lung (WHRIL), a small circular window is implanted in the chest cavity of the mouse (Figure 2E) and permits serial imaging of the same area of the lung for a period of up to 2 weeks (protocol allowance). Using the WHRIL model, the authors were able to image tumor cells within the lung microvasculature (Figure 2F), tumor cell extravasation, cell division, and formation of micrometastases (89). The WHRIL model has capacity to thoroughly characterize the effects of targeted anti-metastatic therapeutics on pulmonary micrometastases and established metastases in a preclinical setting. Using fluorescent reporter genes or functional dyes, in combination with WHRIL model, would permit researchers to assess the effects of gene modulation or targeted therapies on metastatic OS cell biology in the lung, in real-time. The advantages of the WHRIL model include the unprecedented ability to study metastatic OS cells at the cellular, subcellular, and molecular level in live, free-breathing mice. Secondly, serial imaging can be performed to assess the effects of therapy over progressive (albeit limited) time points. One major drawback of this technique is the limited depth of imaging, which in turn is dependent on the type of microscope (single photon vs. multi-photon imaging) and the type of fluorophore used (109). Regular epifluorescence imaging would be limited to an imaging depth of 200 μm due to light scattering. In contrast, using a multi-photon confocal microscope and tumor cells labeled with near-infrared fluorophores (emission wavelengths between 650 and 900 nm) would push the imaging depth toward 700 μm (110). The WHRIL model can be implanted at a timepoint corresponding to tumor cell arrest, extravasation, colonization of extravascular lung tissue, formation of micrometastases, and vascularized macrometastases (steps 4, 5, 6, and 7, Figure 1D).

### Mouse Models of Osteosarcoma Metastasis

Based upon the complexity of metastatic biology, scientific discoveries that lead to new and effective therapies for OS metastases are expected to be derived through experimental models which most faithfully recapitulate the biology and key regulatory pathways involved in the genesis and metastatic progression of OS. Furthermore, models that accurately reproduce the natural progression of spontaneous micrometastases in the absence of a primary tumor are necessary to investigate activities of novel anti-metastatic therapeutics, as this clinical setting is the most pressing scenario in which humans diagnosed with OS require advances in treatment. Although an ideal animal model of OS has yet to be universally recognized or accepted, the most desirable model characteristics should include spontaneous primary bone tumor and pulmonary metastases development within an immunocompetent host.

In whole organisms, such as humans and dogs, successful metastasis occurs only when cancer cells, singly or in groups, become able to dehisce from not only the surrounding normal tissues, but also from malignantly transformed neighboring cells within the primary tumor. To be successful in seeding distant sites, these metastatic precursors must acquire the ability to invade through the tissue matrix, intravasate into the circulation, arrest within the target tissue, extravasate, survive within each of these diverse and heterotypic environments, and then proliferate within the target organ in ways that recapitulate the primary solid tumor (111). Doing so requires the acquisition of myriad behaviors not typical of the cells of origin, and these transformed phenotypes can arise from abnormal activation of cell-autonomous pathways that endow tumor cells with, for example, resistance to apoptosis (112) or the ability to affect unusually high levels of capped mRNA translation (22). Beyond these shifts in behavior that represent intrinsic properties of the malignant cells themselves, disseminated tumor cells often acquire additional malignancy-associated behaviors from interactions with the normal tissues that surround them within the metastatic niche (113). Interestingly, these interactions need not require close contact between the effector cells and the responder cells—they can occur at long distances, even being initiated by cells located within the primary tumor (114).

These complex interactions between malignant tumors and host cells and tissues make the study of metastasis difficult outside of whole organisms. As the laboratory workhorse for most biological systems, murine models have become those that researchers most often use for exploration into the mechanisms of OS metastasis (115). Murine models of metastasis are diverse and can facilitate the study of biology and therapeutic development through manipulation of the host (using genetically engineered mice, or GEMs), manipulation of the tumor cells themselves (using cell lines, xenografts, or allogeneic transplants), or both. Murine systems allow researchers to study elements critical to oncogenesis, as is evident in the multiple GEM models of spontaneous OS (116–118)—even facilitating whole-genome forward genetic screens into mechanisms of oncogenesis and metastasis (119). The use of immunodeficient mice has facilitated a recent explosion in the generation of patient-derived xenograft (PDX) models (120, 121) and their use in OS research (122), including orthotopic models of spontaneous metastasis which mimic the care patients receive through the implementation of hindlimb amputations (123, 124).

**Figure 3:**
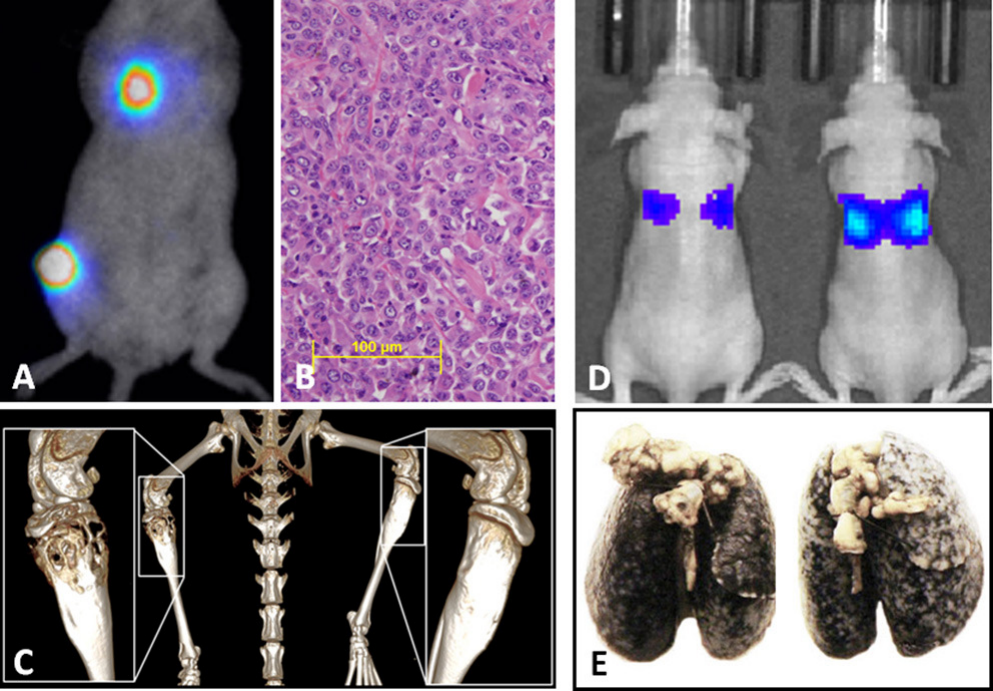
**(A)** Syngeneic orthotopic mouse model of primary bone OS (K7M3) with concurrent spontaneous pulmonary metastases development visualized by bioluminescent imaging, **(B)** with corollary histology of established pulmonary metastatic lesions and **(C)** micro CT images of the OS primary lesion showing profound osteolysis and contralateral unaffected tibia. **(D)** Bioluminescent imaging of an experimental metastases model in athymic nude mice following tail vein injection with the Abrams (canine OS) luciferase cell line demonstrating correlation between luminescent signal and **(E)** gross macroscopic tumor burdens.

Generally, mouse models can be divided into three classes: (1) those that spontaneously develop primary tumors and subsequently develop metastasis, (2) those that are implanted orthotopically (usually into a leg bone) with spontaneous distant metastases (Figures 3A–C), and (3) those where tumor cells are inoculated directly into the circulation (often called experimental metastasis, Figures 3D,E) (115). Each of these approaches can ask different experimental questions, and each has unique strengths and weaknesses that should be recognized when interpreting results and formulating conclusions. Advantages and disadvantages associated with these models are summarized in Table 1.

**Table 1:** Mouse models of osteosarcoma.

	**Advantages**	**Disadvantages**
**TUMOR SOURCE**		
Human cell lines	• Easy to expand • Easy to manipulate genetically • Able to compare across many studies • Those that colonize lung demonstrate tissue tropism	• Serial passage induces genetic and phenotypic drift • Must use immunodeficient mice • Few stable lines available, fewer that colonize murine lungs
Patient-derived xenografts	• Broad panels recapitulate diversity • Better fidelity to original tumor properties/clones • Many stable PDXs available	• Must use immunodeficient mice • Most do not show lung metastasis under traditional conditions • Still questionable retention of original tumor properties/clones
GEM-derived cell lines	• Implantable in immunocompetent mouse strains • High- and low-metastatic cell lines derived without multiple rounds of selection	• Uncertain how well GEM osteosarcoma recapitulates spontaneous disease • Less-well-characterized than human models (genetics/copy number)
Intact GEM mice	• Can engineer to study interplay with genes of interest • Can study earlier stages of malignant transformation	• Patterns of tumor development differ from human (axial/jaw) • Usually multiple primary lesions • Cannot resect/amputate
**MODE OF INTRODUCTION**		
Orthotopic injection	• May preserve original tumor properties/clones • Simple procedure requiring minimal investment in personnel • High take rates in most cell line/PDX models • Can be removed surgically, usually by amputation	• Humane endpoints occur faster and with smaller tumors • Difficult to distinguish procedure-related emboli from metastasis arising from primary tumor
Orthotopic implantation	• Same as for orthotopic injection, except: • Procedure-related tumor emboli unlikely	• Same as for orthotopic injection, except: • More complex procedure requiring large time investment • Lower take rate than for injections • Requires actively growing “donor” tumors
Subcutaneous implantation	• Simple procedure can be high throughput • Many PDX lines already propagated subcutaneously • Can be excised in a simple surgical procedure	• Serial passage in subcutaneous environment introduces phenotypic drift (less than in culture) • Low rates of metastasis from subcutaneous tumors
Intravenous inoculation	• Very high throughput procedure • High rates of metastasis formation in numerous models • Retains tissue tropism to lung • Short time courses for experimentation • Single-step experiments (no resection surgery required)	• Agnostic to early steps in metastasis • Inoculated cells may differ from those that disseminate hematogenously from a primary tumor • Tissue tropism may be weighted toward anatomic circulation patterns and site of injection
**PROCEDURES/MANIPULATIONS**		
Amputation	• Mimics patterns of clinical care in humans and dogs • Allows time for metastases to develop beyond humane endpoint for primary tumor • Mice tolerate procedure and recover well	• Complex procedure requires large investment of time, not high throughput • Morbidities associated with procedure can complicate interpretation
Surgical excision	• Excision of subcutaneous lesions less morbid than amputation • Procedure takes less time than amputation	• Low rates of metastasis from subcutaneous tumors • Adhesions surrounding large lesions can complicate excision

### Three Dimensional Engineered Models of Metastasis

Traditionally, the oncogenic transformation and malignant behaviors of cancer cells have been ascribed to perturbations involving multiple and interactive molecular factors rooted in genetic alterations and dysregulated biochemical signaling. While many aspects of cancer cell phenotype, including metastasis, can be adequately characterized and studied through the singular lens of biology, there is overwhelming evidence that mechanical forces exerted by and upon cancer cells, surrounding stromal elements, and ECM are integrally linked with oncologic activities, including cancer cell invasion and metastasis (51, 125). Living cells are capable of sensing mechanical stimuli (tensile, compressive, and shear forces), termed mechanotransduction, through specialized cellular structures including focal adhesions and stretch-gated ion channels (126, 127), which result in activation of gene and signaling pathways that regulate cellular behaviors. The realization that mechanical cues, in concert with biologic context, contribute collaboratively to diverse cancer processes has spurred rapid advancements in studying cancer metastasis through the deliberate inclusion of physical science, tumor bioengineering, and microfabrication approaches.

While a preponderance of cancer investigations includes studies based on two-dimensional (2D) cell models, such experimental methods that rely upon cancer cells grown in monolayer do not recapitulate the true interactions between cells-cells and cells-extracellular matrices encountered during solid tumor formation, evolution, and metastatic progression. The bidirectional interactions of cancer cells with the tumor microenvironment generates biological complexity, which can be more thoroughly studied through three-dimensional (3D) modeling strategies that include biomimetic engineered tumor models. Through the purposeful design of various mechanical platforms, it is now possible to ask and answer specific questions regarding how cancer cells respond to highly tunable variables including matrix stiffness, interfacial geometry, cell curvature, and other mechanotransduction gradients (128–131). By virtue of precise and reproducible fabrication techniques for generating engineered biomimetics, cancer cell reactivity in response to individual or collective stimuli can be investigated under controllable and quantitative experimental conditions. While providing unique opportunity to study cancer biology, awareness for the strengths and limitations of diverse mechanobiology platforms for elucidating cancer-associated processes is required to ensure their suitable applications. Given their capacity for high throughput data generation, bioengineered 3D cellular platforms are expected to complement existing biologic model systems for rapidly advancing the current state of knowledge regarding cancer metastasis. Several 3D *in vitro* biomimetic platforms currently used in cancer research are summarized, and their suitability for studying unique aspects related to OS metastasis are highlighted.

#### Scaffold-Free 3D Models: Tumor Spheroids

**Figure 4:**
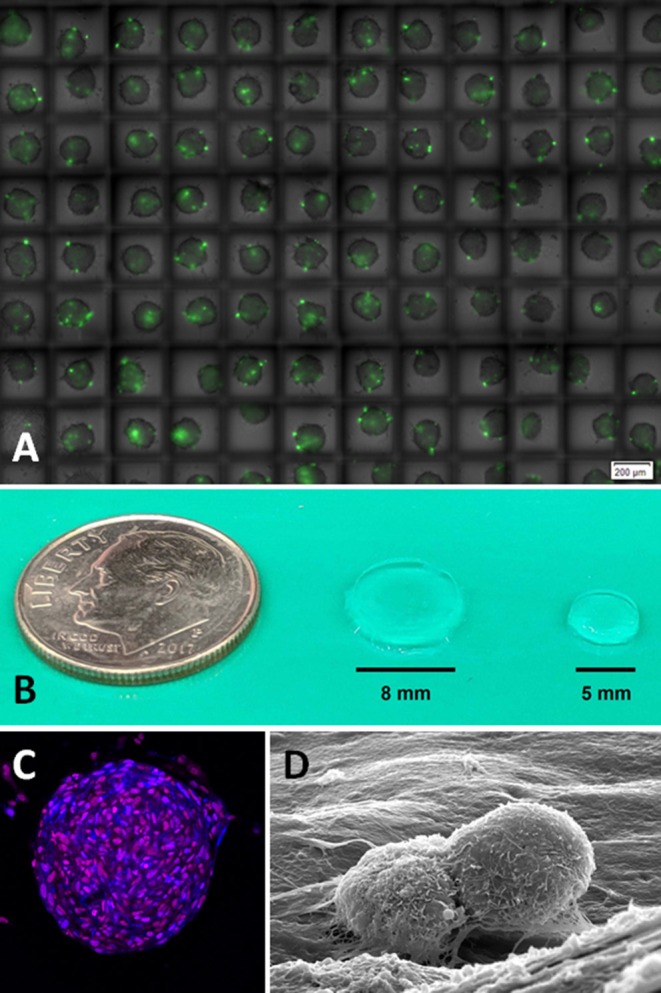
**(A)** Glioblastoma spheroids in high throughput high-density hanging drop culture on a microchip. Method allows for the rapid screening of novel therapeutic agents in cancer cells growing in 3D whereby diffusional gradients and cell-cell interactions are more accurately recapitulated than 2D cell culture conditions (monolayer). Green dye (Celltox™ Promega) shows cell death after 24 h of culture (Image courtesy of Anurup Ganguli and Rashid Bashir, UIUC). **(B)** Relative size of hydrogel scaffolds for the study of **(C)** 3D glioblastoma spheroids by confocal fluorescent microscopy and associated **(D)** homotypic (cell-cell) and heterotypic (cell-ECM) interactions by scanning electron microscopy (Images courtesy of Emily Chen and Brendan Harley, UIUC). UIUC, University of Illinois at Urbana-Champaign.

Tumor cell masses naturally grow in 3D and cellular behaviors are dependent upon multiple biochemical and mechanical cues heterogeneously distributed throughout the microenvironment (i.e., hypoxia and intercellular forces, respectively). Compared to conventional 2D cell culture methods, spherical 3D tumor models are superior for recapitulating the spatial cellular and biochemical heterogeneity of solid tumors. Tumor spheroids are cancer cell aggregates ranging in size from 20 to 1,000 μm in diameter and can be formed through various techniques, with the easiest method reliant upon cell buoyancy (132). Additionally, allowing cells to aggregate by gravity (hanging drop method) or culturing cancer cells on non-adherent or cell-repulsive substrates are alternative strategies for reproducible spheroid formation (133). The simplest spheroid models focus on single cell populations which can self-aggregate and produce endogenous ECM, thereby recapitulating homotypic cell-cell, as well as heterotypic cell-ECM interactions operative during solid tumor formation. The generation of more biologically complex suspension models can be achieved through multicellular spheroids whereby diverse cell populations (cancer, stromal, immune) are intermixed to create more realistic physiologic cues and cellular interactions produced within the naturally occurring tumor microenvironment. Collectively, advantages of tumor spheroid models include high-throughput analysis (Figure 4A) and capacity for rapid scale up, while limitations of scaffold-free 3D spheroids include difficulty in studying more complex and dynamic processes such as angiogenesis, invasion, and metastasis. Based upon these characteristics and limitations, scaffold-free spheroid models are well-suited for preclinical anti-cancer drug screening, characterizing diffusion kinetics and drug resistance mechanisms, and unicellular responses including migration, spreading, ECM deposition, and soluble mediator secretions (133, 134).

Specific for OS, 3D culture systems with spheroids have been utilized for the past 2 decades for studying the effects of the tumor microenvironment on various aspects of OS biology and has been thoroughly summarized by De Luca et al. (135). Derived from these multiple investigations and relevant to therapeutic strategies specifically for OS metastasis, OS spheroids have shed illumination on drug resistance mechanisms to conventional chemotherapeutics (136–142), the maintenance of cancer stem cells and tumor-initiating cells (139, 143, 144), impact of ECM stiffness and composition on metastatic phenotype (145, 146), cues that promote vasculogenic mimicry (147, 148), and metastasis favoring pathways including the roles of specific transcription factors (NF-κB) (149, 150) and miRNAs (151). In addition, the feasibility of generating co-culture bicellular spheroids through the combination of HUVEC and MG-63 cells for the study of VEGF-mediated angiogenesis has recently been described (152).

#### Scaffold-Based 3D Models

The ECM is critical in shaping tumor biologic responses through mechanotransducive mechanisms, and the investigation of cancer cells embedded within scaffold-based constructs that vary in chemical composition, shape, density, structure, and porosity allows for researchers to dissect differential mechanotransductive contributions for the induction of diverse malignant phenotypes and cellular processes displayed by cancer cells. Scaffolds can be constructed from either natural or synthetic polymers, with both sharing conserved properties of biocompatibility and promotion of cellular adhesion. Natural scaffold materials are typical ECM proteins while synthetic scaffolds are derived from tunable and crosslinking materials including polyethylene glycol (PEG) and polylactide-co-glycolide acid (PLGA), as well as porous ceramic biomaterials such as bioactive hydroxyapatite and tricalcium phosphate.

Hydrogel scaffolds (Figure 4B), composed of natural or synthetic polymers, are widely used for studying biologic responses of cancer cells, as a gel medium mimics the natural *in vivo* microenvironment of nascent tumor mass growth in 3D (Figure 4C), whereby cell-to-cell and cell-to-matrix interactions are preserved for directing phenotypic behaviors including proliferation, migration, chemoresistance, and angiogenesis (153, 154). The most common biocompatible polymeric hydrogel materials include collagen type I, Matrigel, and alginate; and these natural materials facilitate cancer cell attachment through heterotypic interactions via integrin receptors and ECM which regulate cell survival, growth, and differentiation (Figure 4D). In addition to natural biomolecules, synthetic constituents used for hydrogel formulation can include polyethylene glycol, polylactic acid, polyglycolic acid. By virtue of their chemistry, synthetic hydrogels have the advantage of being chemically tunable (stiffness, porosity, adhesion ligand density) via synthesis or crosslinking (155), and can recapitulate spatiotemporal changes in matrix heterogeneity encountered within the tumor microenvironment. Increasing sophistication of hydrogel-based models can be achieved through a combination of chemical engineering and biologic layering, including the construction of soluble mediator (growth factors, chemokines, peptidyl signaling molecules) gradients or combinatorial co-culturing of cancer cells with stromal cells including endothelial cells, fibroblasts, and immune cells. While hydrogels have been explored as a controlled drug release scaffold strategies for OS therapy (156–158), the study 3D scaffold tumor models for unraveling OS biology and metastasis remains limited, with some investigations describing differences in behavioral phenotype of malignant OS cells compared to non-transformed osteoblasts based upon matrix rigidity and elasticity (159, 160). In addition to hydrogel scaffolds, chitosan, silk, and synthetic polymers have served as adhesive constructs for 3D OS modeling and have illuminated mechanisms behind viral permissiveness (161), hypoxia-induced angiogenic mediator secretions (162), drug resistance (163), and maintenance of stem cell phenotype (164).

#### Microfluidic Platforms: Organ-on-a-Chip

While 3D spheroids with or without scaffolds provide valuable information on cell-cell and cell-ECM interactions, the static nature of nutrient and metabolic waste transport under typical 3D culture systems does not accurately replicate spatiotemporal diffusional gradients naturally formed from lymphatic or blood vessel formation within solid tumors. Microfluidic systems are precisely fabricated from molds and made of materials that are biocompatible, oxygen permeable, and tunable (stiffness, temperature, shear flow pressure, molecular gradients). Structurally, microfluidic systems can be fabricated to include diverse shapes on a micro- or nanoscale including channels and chambers with highly precise diameters, shapes, and flow control rates. When combined with 3D cell culture systems such as spheroids, microfluidic platforms can recapitulate diverse complex processes representing different stages of the cancer progression including tumor-vascular interface responses, diffusional effects of biomolecules on cell populations, and pathologic cancer processes including invasion, angiogenesis, and metastasis (165–170). Recently, specific metastasis-on-a-chip platforms have been fabricated allowing for real time tracking of fluorescently labeled cancer cells and their heterotypic interactions with both ECM and normal resident cells along the full continuum of the metastatic cascade (171, 172). Kong and colleagues recently reported the construction and use of a microfluidic platform for studying the organotropism of cancer cell metastasis and demonstrated the correlative value of their microfluidic system with athymic nude mice models for the evaluation of small molecule inhibiting anti-metastatic strategies (173). Specific for OS, 3D microfluidic platforms have been used to study OS cell adhesive properties under various physiologic conditions (pH, temperature, shear flow) (174), cell morphology in response to gradient molecules (175), and drug screening of nanoparticle encapsulated chemotherapeutics (176).

### Spontaneous and Immunocompetent Dog Model of Metastasis

Conventional OS models for studying experimental therapies most frequently are reliant upon xenogeneic and syngeneic transplant models conducted in mice, however, the inclusion of complementary model systems (CAM, PuMA, WHRIL, engineered 3D biomimetics) have gained wider appeal and scientific acceptance for improving predictive modeling of cancer biology and metastasis. While xenogeneic models, including patient derived xenografts, may provide information pertaining to the sensitivity of human OS tissues or cell lines to specific therapeutics, tumor-host interactions (especially immunobiologic responses) are poorly recapitulated in comparison to what occurs in people who develop OS spontaneously. Although syngeneic models more accurately represent immunologic tumor-host responses than xenogeneic systems, the process of tumor formation and spontaneous metastasis in any transplant model remains artificial, likely underestimating the complexity for how OS naturally progresses in an immunocompetent host. To accurately identify and expedite the clinical translation of novel therapeutics to people with metastatic OS, the evaluation of experimental strategies, in particular immune-based, should be conducted in the most highly relevant and immunocompetent tumor model.

Besides people, canines are the only other large mammal that spontaneously develops OS with substantive frequency. Canine appendicular OS is the most common primary bone tumor in dogs of large to giant skeletal size, and has been estimated to affect at least 10,000 pet dogs every year in North America (58), which is 10 times greater than the number of pediatric OS patients diagnosed annually in the United States. The clinical presentation, biologic behavior, natural disease progression, and genetic signature of OS in dogs is similar to people (177–179), and collectively emphasizes the comparative relevance of dogs to serve as a model system for both discovery and therapeutic investigations (180–184). This modeling strategy has been advocated by leaders in the field of OS basic science and clinical research, and ascribes value on the inclusion of pet dogs with OS as a distinctively informative model system for prioritizing novel therapeutic agents that target metastatic progression (8).

## Standard of Care and the Unmet Need for New Anti-Metastatic Therapies

The current standard of care for human patients diagnosed with OS remains largely unchanged from that first used in the early 1980s (185), being neoadjuvant and adjuvant MAP chemotherapy (methotrexate, doxorubicin, cisplatin) together with aggressive local control by surgical excision (186). Building on techniques pioneered in pet dogs with OS (187, 188), most human patients diagnosed today benefit from limb salvage reconstructive techniques that preserve limb function. With these standard of care therapies, outcomes for patients with localized disease increased markedly, such that up to 60% of patients experience “cure” (5-year event-free survival) (189). However, these outcomes have changed little over the last four decades (190).

The factor that most strongly influences outcomes in human patients is the presence or absence of metastatic lesions, usually of the lung parenchyma. Patients who develop lung metastases, whether at diagnosis or years after completing therapy, face a dismal prognosis, with fewer than one in five patients surviving more than 5 years beyond this event (189, 191). Multiple efforts to improve this outcome through intensification of systemic therapy or the introduction of novel regimens have not succeeded.

Patients with both resectable and unresectable metastatic disease at relapse are usually offered systemic therapy, most commonly with high dose ifosfamide (192) or multi-tyrosine kinase inhibitors (193). Although these therapies do little to effect long-term outcomes, they can facilitate short-term disease control and prolong survival. While radiation has a relatively minor role in the curative care of patients with either localized or metastatic disease, modern techniques can be extremely helpful in the palliative setting, providing excellent disease and symptom control (194). The only intervention proven to offer hope for long-term “cure” of disease in patients with metastases remains surgical excision of all macrometastases, and several studies suggest that up to 30% of patients who achieve complete surgical remission will survive disease-free beyond 5 years (195–197).

## Novel Therapeutic Strategies for Combating Pulmonary Metastases

### Tumor-Specific Molecular Vulnerabilities

As mentioned previously, each step of the metastatic cascade is a rate limiting step. For example, if a new drug can prevent OS cells from leaving the primary tumor, invading local tissue, or entering local blood vessels, then the metastatic cascade is stopped in its tracks. Indeed, every step of the metastatic cascade harbors several druggable targets in various types of cancer, as summarized in Table 2. In the clinical setting, however, it is presumed that patients with localized tumors already have subclinical micrometastatic disease in the lung. Thus, targeted therapies that act within the microenvironment of the primary tumor may not necessarily be effective on tumor cells have already spread to the lung since adaptation strategies depend on the particular microenvironment the tumor cells reside. In this scenario, therapeutic strategies that target the processes involved in lung colonization and micrometastases formation would be expected to be most effective in delaying metastatic progression. Further basic research into the molecular pathways underpinning OS lung colonization, micrometastases formation, and the establishment of macrometastases is needed to uncover more actionable targets.

**Table 2:** Druggable molecular targets in the metastatic cascade.

**Step of the metastatic cascade**	**Actionable target(s)**	**Inhibitors**	**Inhibit lung metastasis in preclinical model? (cancer type)**	**References**
Migration, intravasation	PAK1	IPA3	Yes (ESCC)	(13)
Intravasation	uPAR	SRSRY	Yes (OS)	(14)
Transit within blood	TDO2 αvβ3	680C91 IH1062	Yes (BC) Yes (Mel)	(15) (16)
Extravasation	VCAM α5β1 CCR2	α-VCAM Ab PHSCN TC1-TSL	Yes (Mel) Yes (BC) Yes (Mel, Col)	(17) (18) (19)
Lung colonization	GRP78 CDK12/13 BRD4 mTOR Ezrin HDACs PKC IL-6ST CXCR1/2 PD-1/Lag-3/ NK activity	IT-139 THZ531 JQ1 Rapamycin NSC305787 NSC668394 MS-275 (Entinostat) UCN-01 sc-144 DF2156A α-PD-1, α-Lag-3 Abs IL-2	Yes (OS) Yes (OS) Yes (OS) Yes (OS) Yes (OS) Yes (OS) Yes (OS) Yes (OS) Yes (OS) Yes (OS) Yes (BC)	(12) (20) (21) (22) (23) (23) (24) (25) (26) (26) (27)
Micrometastases	Cell surface- GRP78 PD-1	BMTP-78 Anti-PD-1 mAb	Yes (BC) Yes (OS)	(11) (28)
Macrometastases^*^	Procaspase-3	PAC-1	Yes (OS)	(29)

* *Studies using an animal protocol where treatment was given after establishment of lung metastases. OS, osteosarcoma; BC, breast cancer; Mel, melanoma; Col, colon cancer; ESCC, esophageal squamous cell carcinoma*.

### Targeting the Tumor Microenvironment

Successful dissemination and colonization of distant tissues by a tumor cell requires navigating a gauntlet of interactions with normal cells and associated tissues (114). Each interaction can either help or hurt that cancer cell's chance of survival. The striking tropism that OS displays for lung tissues suggests that tumor cells elicit or receive signals from cells within the lung metastatic niche that facilitate their survival. Several emerging studies have defined characteristics of that environment that might support tumor growth, many of which constitute targetable vulnerabilities, including pathways that promote dormancy, alter susceptibility to chemotherapy, facilitate metastatic outgrowth, and the maintenance of stemness.

Stromal elements produced by both host cells and tumor cells may play a particular role in the survival of metastasis-initiating cells and in the maintenance of their stem-like features. Zhang and colleagues recently showed that FGF signaling within the metastatic environment triggers a fibrogenic program within disseminated tumor cells that promotes their stemness and survival (198). Signals transduced by way of mTOR complex 1 initiate this program, although the subsequent production of fibronectin by OS cells can then maintain this stem-like state independent of host signals, including FGF. These studies stop short of testing the therapeutic potential of targeting these pathways, but existing agents should facilitate future assessment of their capacity to affect disease progression, most likely in preventing emergence of late metastases.

The laboratories of Roberts and Cam have identified targetable bi-directional signaling between OS cells and lung epithelial cells that appears critical for metastatic colonization. Using a combination of human tissues, xenografts, syngeneic mouse models, and canine models of disease, they have shown how a ΔNp63/IL6/CXCL8 signaling axis mediates tumor-host signaling events critical to the metastatic process. In their model, tumor cells primed by aberrant expression of ΔNp63 (112) (an alternative isoform of the p53 family member TP63) respond to signals from lung epithelial cells by producing high levels of IL6 and CXCL8 (199). Disruption of these cytokine/chemokine signals effectively reduced metastasis formation. Indeed, more than 80% of mice treated with inhibitors of both IL6 and CXCL8 signaling survived long term, while 100% of mice bearing the same tumors succumbed to metastatic disease (26). Interestingly, this antimetastatic effect was only achieved with combination therapy. Mice treated with one or the other inhibitor showed only modest inhibition of metastasis, suggesting some signaling pathway redundancies that remains undefined. Unfortunately, inhibitors that proved effective in their models are unlikely to be developed clinically. Work aimed at identifying critical signaling nodes up- or down- stream of these pathways may identify targets that are more effective and druggable with small molecule inhibitors well-suited for clinical implementation.

Signals that facilitate tumor cell survival within the metastatic niche can emerge from either lung-resident cells or from cells that invade that niche, often in response to tumor-derived signals. For example, Baglio and colleagues have shown that TGFβ expressed on the surface of extracellular vesicles from OS cells can also elicit production of large amounts of IL6 by mesenchymal stem cells (200). The release of this cytokine into the metastatic niche triggers activation of STAT3 within the tumor cells, which promoted proliferation of those metastatic cells in their models. In evaluating the therapeutic relevance of this phenomenon, they showed that the administration of anti-IL6 antibodies reduced the number of metastatic lesions that formed in their animals (200).

Some tumor-host interactions prove detrimental to the survival of disseminated tumor cells. Kleinerman's group has made a series of observations that suggest most disseminated tumor cells that reach the lung will be eliminated through activation of a suicide signal when the FAS receptor expressed on the surface of the tumor cells engages FAS ligand, which is expressed constitutively within the lung (201). This phenomenon results in the selection of a subpopulation of tumor cells that are FAS-negative (202). Interestingly, they have shown that FAS downregulation within this subset of malignant cells can be reversed, as exposure to inhaled gemcitabine drives re-expression of the FAS receptor, engaging the death-inducing signaling complex and triggering apoptosis (203). Such therapies have yet to be tested clinically in pediatric OS patients but seem viable. As proof-of-concept, a study by Rodriguez and colleagues demonstrated that pet dogs with macrometastatic pulmonary OS receiving treatment with aerosolized gemcitabine did result in the upregulation of FAS receptor and markers of cell death by OS cells within pulmonary metastatic lesions (204).

Searching for epigenetic changes that facilitate metastatic colonization of lung tissue by OS cells, Morrow and colleagues recently identified genetic loci that acquire enhancer activity in cells with high metastatic potential (21). Among genes regulated by these metastatic variant enhancer loci, Factor 3 (F3, a gene which can activate blood clotting) demonstrated particular importance for metastasis when evaluated functionally. Disruption of F3 production by OS cells significantly impeded metastatic colonization efficiency in animal models but did not affect primary tumor growth (21). Interestingly, the importance of blood clotting for lung colonization in OS may have been suggested in previous work, lending credence to these findings (205, 206). While this target has not been evaluated in a therapeutic setting, F3 signaling (which triggers both clotting and intracellular signal cascades) should be targetable using existing, FDA-approved drugs (207).

### Potential Metabolic Vulnerabilities of OS

The unique metabolic demands of the primary tumor vs. metastasis are reflective of their different microenvironments (cellular and extracellular components), nutritional availabilities, and level of oxygenation. A rapidly growing primary tumor mass requires a constant supply of energy (ATP) and biomacromolecules (lipids, carbohydrates, and proteins) (208). During glycolysis in normal cells, ATP is obtained from glucose via the oxidation of its carbon bonds through mitochondrial respiration, a process which also requires oxygen. However in cancer cells, the glycolytic intermediate pyruvate is shuttled away from the tricarboxylic acid cycle, and is fermented into lactic acid, even in the presence or absence of oxygen—a phenomenon called the Warburg effect; and several theories on how the Warburg effect might benefit proliferating cancer cells has been discussed elsewhere (209). Not surprisingly, subversion of the Warburg effect has been observed in several OS cell lines such as LM7 and 143B (210). Furthermore in a preclinical mouse model, Hua and colleagues demonstrated LM8 tumor-bearing mice had elevated levels of serum pyruvic acid and lactic acid compared to healthy controls, which suggested that proliferating OS tumor cells were highly glycolytic. The serum from tumor-bearing mice also had higher levels of intermediate metabolites of the tricarboxylic acid cycle compared to healthy controls, further underscoring the higher energy demands of proliferating OS cells within localized and metastatic sites (211). Interestingly, the majority of circulating metabolites in serum were lowest at initial primary tumor formation (week 1) and again at metastatic progression (week 4) following LM8 inoculation, which could suggest that similar global metabolic transformation mechanisms were shared by OS cells during incipient primary tumor growth and distant metastases development. Mechanistically, Hua and colleagues suggested that the unexpected lower metabolic profile in tumor-bearing mice identified at week 4 (metastatic progression) may be due to tumor microenvironmental hypoxia that restricted OS cell growth and reduced cellular metabolism, although this possibility wasn't confirmed in their study (211).

Surviving in the lung microenvironment presents a unique set of metabolic challenges that are distinct from the primary tumor. As mentioned previously, redox stress appears to be a major microenvironmental stressor in the lung. Reactive oxidative species (ROS) and reactive nitrogen species (RNS) produced by the lung parenchyma can affect tumor cell mitochondrial function in a number of ways (58, 212). For example, it is generally known that excess ROS, such as superoxide (O_2_^−^), can modify mitochondrial DNA, which in turn, can negatively affect the electron transport chain (ETC), mitochondrial membrane potential, and ATP production (213). Prolonged exposure to RNS such NO^−^ can irreversibly inhibit complex I of the ETC (214). Peroxynitrite (ONOO^−^), another potent RNS, can inhibit multiple enzymes in the mitochondria such as complexes I–IV, as well as aconitase of the tricarboxylic acid cycle (214). Metabolic adaptation to such oxidative stress would be a pro-survival phenotype that would be selected for during the colonization process, and not surprisingly, anti-oxidant responses which consists of either the upregulation of redox-related enzymes or altered glutathione (GSH) metabolism have been observed in metastatic breast (63), melanoma (61), and osteosarcoma (21, 215). For example, Ren and colleagues have found that metabolites in the GSH metabolic pathway were found to be significantly altered in highly metastatic OS cells compared to their clonally related, low metastatic counterparts (215). Shuttling of metabolites into the GSH pathway is important for producing GSH, which in turn, react with and neutralize ROS and RNS to form less reactive intermediates (216, 217). Other metabolic pathways that were found to be altered in highly metastatic OS cells include arginine, inositol, and lipid metabolic pathways. The previously mentioned study by Hua and colleagues found that serum metabolites of lipid metabolism were found to be elevated in mice with lung metastases compared to tumor-bearing mice with no metastases (211). These observations noted by Hua and colleagues in a mouse model of OS are congruent with global lipidomic studies identifying differences between metastatic (143B) and non-metastatic (HOS) human OS cell lines (218), as well as the recognized importance of lipid metabolism in cancer metastases (219). Collectively, derived from preclinical studies inclusive of cell lines and murine models of cancer, evidence supports altered lipid metabolism being important for metastasis progression; where increased lipid production may address the heightened demand for membrane synthesis during cell growth and organelle biogenesis. As such, targeting unique metabolic demands of metastasis offers a new avenue of anti-metastatic therapy. Indeed, Ren and colleagues demonstrated that targeting the inositol metabolism of metastatic OS cells prevented their growth in the lung microenvironment (215). Further studies are needed to elucidate whether other metabolic susceptibilities exist in metastatic OS, and whether these metabolic susceptibilities can be exploited for new therapeutics.

### Leveraging the Immune System to Combat OS Metastases

Recently, immunotherapy has been heralded as a breakthrough for the management of diverse liquid and solid tumors, and its ascension as a major therapeutic pillar is underscored by a rapidly increasing number of FDA approved immune-based treatments for cancers that are resistant to conventional modalities. The anticancer activities of immunotherapies can be ascribed to the cooperative effector functions exerted by both the innate and adaptive immune arms, and while immunotherapy is highly effective for certain solid tumors like melanoma, renal cell carcinoma, and others, its promise for benefiting patients diagnosed with metastatic OS remains largely disappointing to date (220–223). Paradoxically, there is convincing evidence that OS can be recognized by trafficking immunocytes, yet successful exploitation of immunotherapeutic strategies remains elusive. To accelerate the clinical deployment of effective antitumor immune approaches for combating OS, recent scientific investigations have focused on characterizing the quantity, phenotype, dynamics, and functional nature of immune cells that infiltrate into primary and metastatic OS lesions, and these collective findings have been recently and thoroughly summarized (223, 224).

By way of detailed analyses, several innate and adaptive immunocytes have been identified to putatively participate in the initiation or suppression of anti-OS immune responses and include a plethora of diverse myeloid and lymphoid cell types. Of the various immunocytes identified within the OS microenvironment, both innate affector and adaptive effector populations have been characterized, and include antigen-presenting cells (macrophages/dendritic cells) and T lymphocytes, respectively. Within primary OS lesions, tumor-associated macrophages (TAMs) that can be distinguished via genomic signatures, cell surface markers, and functional activities (inflammation vs. immunosuppression) have received considerable attention for their prognostic value and functional role in OS metastasis (225–230). Most, but not all, investigations have identified that increases in TAMs (quantity) or macrophage infiltrate profiles (quality) favoring a M1-subtype polarization (INOS^+^; pro-inflammatory) rather than a M2-subtype profile (CD163^+^; immunosuppressive) are associated with better overall survival in OS patients. Incongruent findings among studies regarding the role of TAMs in OS biology could be related to the inherent limitations of single timepoint tissue assessments which fail to capture the dynamic nature of immune cell infiltration within the tumor microenvironment. Nonetheless, the majority of histologic findings provide supportive justification to therapeutically manipulate TAMs profiles within OS lesions that have potential to either favor immune activation (Mifamurtide) (231) or inhibit M2-macrophage polarization (ATRA) (232, 233), for the intended purpose of inhibiting metastatic progression.

Complementing the participatory role of TAMs, several studies have focused on characterizing tumor infiltrating lymphocytes (TILs) and their contribution to metastasis immunobiology. Analyses of TILs within the OS microenvironment have shown that both effector and suppressor T lymphocyte (CD3^+^) phenotypes participate in shaping immunosurveillance of OS lesions (229, 230, 234–236). Furthermore, several studies suggest that the density (number) or phenotype (activated or exhausted) of effector TILs within OS primary tumors correlate with prognosis. With regards to TILs density, recent studies have demonstrated that increases in the absolute number of CD8^+^ TILs or the ratio of CD8^+^/Foxp3^+^ TILs significantly correlate with improved overall survival (230, 234). Provocatively, the functional relevance of TILs and operative checkpoint blockade mechanisms might be especially important for metastases, as some studies have found the density of TILs to be enriched in metastatic lesions compared to primary tumors (236, 237). Despite the presence of TILs within OS lesions, several studies suggest that the activity of effector TILs might be attenuated, as supported by the expression of exhaustion markers (PD-1, CTLA-4, Tim3) by TILs and/or tumoral microenvironmental expression of PD-L1 (227–229, 235–237). Collectively, these detailed studies strongly suggest that OS lesions can be effectively infiltrated by T lymphocytes, and that therapeutic modulation of checkpoint blockade strategies could improve TILs effector capabilities.

With a basal understanding for the collection of immunocytes that are present within primary tumor and metastatic OS lesions, rational design of immunotherapeutic interventions can be constructed. Through these concerted efforts, the scientific and clinical oncology community can continue to forge toward understanding fundamental anticancer immune mechanisms and improving treatment outcomes in patients with OS metastases through diverse immunologic strategies, either singly or in combination with conventional therapies (radiochemotherapy).

#### Immune Modulators

Immunomodulatory agents modify immune responses by amplifying the recognition of cancer cells (immunostimulation) or by attenuating the immunosuppressive activities exerted by cancer cells within the local tumor microenvironment. The innate arm of the immune system comprised of natural killer cells, macrophages, dendritic cells, and primordial T cell subsets (natural killer and γδ) are predominant effector targets of immunomodulatory strategies. The clinical significance of immunomodulatory interventions relevant to sarcomas was noted over a century ago, when William Coley in 1891 reported objective responses in a small minority (10%) of patients with non-resectable sarcomas (bone and soft tissue) treated with heat-inactivated *Streptococcus pyogenes* and *Serratia marcescens* injections, termed Coley's toxin (238). The potent anticancer activities induced by bacterial products noted by Coley have been corroborated in both canine and human OS patients that develop surgical site infections (188, 239, 240), and mechanistically these favorable immunologic effects have been attributed to toll-like receptor activation with consequent amplified macrophage and natural killer cell effector functions in mouse models of OS (241).

The clinical translation of immunomodulatory agents which stimulate the innate immune arm for improving outcomes in OS patients remain limited, but include liposome-encapsulated muramyl tripeptide phosphatidylethanolamine (L-MTP-PE) and cytokine-based therapies. Based upon its mechanism *in vitro* and in preclinical investigations for activating monocytes and macrophage to a tumoricidal state (242, 243), as well as its unique evaluation singly or in combination with cisplatin in pet dogs with OS (243, 244), clinical investigations of MTP-based strategies have been conducted prospectively by the Children's Oncology Group consortium. In a seminal study by Meyers and colleagues, the addition of MTP to a MAP (methotrexate, doxorubicin, cisplatin) backbone in patients with localized OS significantly improved 6-year overall survival rate from 70 to 78% (245). Additionally in the setting of metastatic and/or recurrent OS, the 5-year event free survival rate of patients receiving chemotherapy alone (26%) vs. chemotherapy with L-MTP-PE (42%) appeared favorable (246), further supporting the clinical benefit of this immunomodulatory strategy for delaying the natural progression of OS pulmonary metastases. Complementing the mechanism of L-MTP-PE, exogenous cytokine therapies have also produced marginal improvements in patients diagnosed with OS. In particular, INF-α-2b and IL-2 have been evaluated in the adjuvant setting with either chemotherapy or other immune-based strategies. Recently, the 3-year event free survival benefit derived from adjuvant pegylated INF-α-2b with MAP has been described in a large consortium trial (EURAMOS-1) (247). While early results have not demonstrated significant improvements in event free survival between MAP alone (81%) vs. MAP with adjuvant pegylated INF-α-2b (84%), long term follow up remains active and will ultimately determine if adjuvant pegylated INF-α-2b has any definitive immune activating role for improving the control of OS micrometastases.

In the setting of macroscopic OS metastases, the tolerability and potential benefit exerted by exogenous IL-2 has been explored. In one study, Meazza and colleagues reported the outcomes of 35 pediatric OS patients with macroscopic OS treated with surgery and combinatorial chemoimmunotherapy comprised of IL-2, MAP, ifosfamide, and lymphokine-activated killer (LAK) cell infusion. While the study was not designed to determine the immunobiologic benefit derived from IL-2 and LAK cell infusion, adverse effects associated with IL-2 therapy were tolerable (grade I and II) with most common side effects being fever, flu-like symptoms, hypotension, and cytokine release syndrome (248). In a different study, Schwinger and colleagues reported the tolerability and activity of single-agent, high-dose IV IL-2 therapy in 10 pediatric patients, in which 4 adolescents had metastatic OS (249). While 2 of 4 OS patients achieved complete remission for 14 and 42 months in duration, systemic toxicity associated with high-dose IV IL-2 therapy was significant with 60–100% of treated patients experience some form of grade III or IV clinical toxicity (fatigue, anorexia, or diarrhea). Despite the high level of toxicity, this study clearly demonstrated the potential for IL-2 to amplify anticancer immune responses sufficient to regress macroscopic OS burdens. In attempts to reduce the toxicity associated with systemic IL-2, yet maintain favorable anticancer immune activities within the anatomic site of metastases (lungs), two significant studies have been conducted in pet dogs with pulmonary metastatic OS, which leverage innovative drug delivery or site-specific gene transducing strategies. Khanna and colleagues evaluated the feasibility and activity of aerosolizing liposome encapsulating IL-2 in pet dogs and demonstrated that robust anticancer immune effects could be induced within the pulmonary parenchyma sufficient to cytoreduce macroscopic OS burdens (2 of 4, CR) without significant toxicity (250). A complementary study reported by Dow and colleagues investigated the activity of intravenously administered liposome-DNA complexes (LDC) encoding the IL-2 gene in dogs with macroscopic OS metastases (251). Infusions of LDC was well-tolerated, generated systemic immune activation, and transgene IL-2 expression within the lung parenchyma. Furthermore, objective cytoreductive activities (2 PR, 1 CR) were achieved in three of 20 dogs treated.

#### Monoclonal Antibodies

The engineering of monoclonal antibodies to enhance the immune system's attack on cancer cells has dramatically expanded therapeutic options for multiple hematopoietic and solid tumor histologies, and currently over a dozen antibodies have received FDA approval for treating different cancers (252). Upon binding to their cognate epitope, monoclonal antibodies exert anticancer activities through various methods including direct cell killing, immune-mediated cell killing (phagocytosis, complement activation, or antibody-dependent cellular cytotoxicity), or disruption of the tumor microenvironment through vascular and stromal cell ablation. While widely instituted and capable of dramatically improving survival outcomes in patients diagnosed with specific forms of hematopoietic cancers, the clinical impact of monoclonal antibodies remains more limited for solid tumors, and almost non-existent in aggressive sarcomas. In part, the restricted application of monoclonal antibodies for OS metastases therapy is driven by the limited expression of extracellular membrane epitopes, as underscored in the study reported by Ebb and colleagues whereby adjuvant trastuzumab (HER2 targeting antibody) combined with chemotherapy failed to improve outcomes in patients diagnosed with metastatic OS (253). Nonetheless, several OS expressing epitopes remain a focus of interest for improving the management of OS metastases, and include GD2, GPNMB, and RANKL (254–257). In particular, GD2 as a surface epitope on OS cells continues to be an actively explored target with several ongoing clinical trials evaluating various anti-GD2 antibody strategies.

In addition to monoclonal antibodies that target OS cells, considerable focus has been on manipulating tumor-specific or tumor microenvironmental (TME) cues with antibody strategies, specifically enhancing the quantity and quality of intratumoral infiltration with immune cell populations through blockade of checkpoint signaling (258). Checkpoint blocking antibodies have been a breakthrough for the management of diverse cancer types including melanoma, non-small cell lung cancer, renal cell carcinoma, bladder cancer, and head and neck cancers. For these collective solid tumors, blockade of immune checkpoints, including CTLA-4, PD-1, and PD-L1, with antibodies have provided life-saving anticancer activities to a subset of patients who would otherwise experience disease progression and death. While several pieces of basic and clinical evidence support the potential benefit of checkpoint inhibition for the treatment of OS metastases including mutational burden, neoantigen load, overexpression of PD-L1 by OS cells and tissue samples, and preclinical activity of checkpoint inhibition in mouse models of metastatic OS (259–262), the clinical activity of single- or combined- checkpoint blockade in treating patients with advanced OS have been largely unfavorable, with objective response rates (CR or PR) ranging from 0 to 5% (NCT011445379, Ipilimumab; NCT02500797 [Alliance A091401], Nivolumab + Ipilimumab; NCT02301039 [SARC028], Pembrolizumab) (263). Given these initial disappointing results, combination therapies have been proposed whereby small molecule agents (Nab-rapamycin, apatinib, axitinib) that target multiple signaling pathways (mTOR, VEGFR1, VEGFR2, PDGFRβ, c-kit) should be combined with checkpoint blockade antibodies in hopes of improving response rates (264).

#### Vaccines

Tumor vaccines are an active form of immunotherapy in which robust adaptive immunity is developed and cytotoxic T lymphocytes are generated for recognizing and killing cancer cells. The induction of antitumor responses against known or unidentified tumor antigens can be achieved through vaccine strategies inclusive of whole cells, lysates, proteins, DNA, RNA, or peptides. For vaccine strategies to be effective, dendritic cells must present tumor peptides within major histocompatibility complexes, as well as costimulatory signals, to fully activate the effector functions of cytotoxic T lymphocytes. Given the key role of dendritic cells for initiating the activation and proliferation of cytotoxic T lymphocytes with anticancer effector functions, dendritic cell vaccines have been explored in patients with metastatic OS through the conductance of two recent clinical studies (265, 266). Miwa and colleagues evaluated 14 patients with recurrent and/or metastatic OS who were treated with 6 weekly subcutaneous vaccinations with autologous dendritic cells pulsed with autologous tumor lysate, TNFα, and OK-432 (265). While treated patients did demonstrate systemic immune activation represented by increases in circulating INFγ and IL-12, none of the OS patients achieved an objective response to dendritic vaccination alone. These negative findings were consistent with an earlier study in which Himoudi and colleagues evaluated 13 patients with relapsing OS treated with intradermal vaccines of autologous dendritic cells matured with autologous tumor lysate and keyhole limpet hemocyanin (266). While the vaccination protocol was well-tolerated, tumor-specific immune activation assessed by the identification of INFγ secreting T cells (ELISPOT) was only observed in 3 patients, and none of the OS patients experience any measurable reduction in macroscopic tumor burden. In light of these initial disappointing studies with dendritic cell vaccination in OS patients, ongoing efforts are focusing on innovative combinatorial strategies to boost OS cell antigen expression, as well as attenuate the localized immunosuppression associated with the tumor microenvironment.

Recently, a vaccine strategy based upon the intravenous infusion of a genetically modified, live attenuated *Listeria monocytogenes* which expresses three immunodominant epitopes of HER2 has been evaluated for in patients with HER2-expressing solid tumors (NCT02386501). This vaccine construct (ADXS31-164) was granted Fast Track designation by the FDA for treatment of patients with newly diagnosed, non-metastatic, surgically-resectable OS, and patients will be treated with this vaccine strategy through the Children's Oncology Group consortium. As a predecessor to the pediatric trial, ADXS31-164 was piloted in pet dogs with OS and provided valuable translational information. Mason and colleagues demonstrated that treatment with ADXS31-164 induced HER2-specific immunity in 15/18 dogs and resulted in a significant increase in median disease-free interval (615 days) and median survival (956 days) when compared to a historical control group. Overall survival rates at 1, 2, and 3 years for dogs treated with ADXS31-164 were 78, 61, and 50%, respectively (267).

#### Adoptive Cell Therapies

Adoptive cellular therapies involve the direct administration of either innate (natural killer cells) or adaptive (cytotoxic T lymphocytes) immune effector cell populations that have been genetically manipulated. The infusion of cellular therapies, specifically cytotoxic T lymphocytes into tumor-bearing patients, circumvents the reliance of vaccine strategies to successfully active a large population of tumor-specific effector lymphocytes in the recipient host. For T-cell based strategies, adoptive cell therapies rely upon the genetic engineering of T lymphocytes to either express a known T cell receptor (transgenic TCR) or chimeric antigen receptor (CAR), with both strategies resulting in the production of T cells with defined specificity, which can be MHC-restricted (transgenic TCR) or -unrestricted (CAR). While CAR technology has clinically impacted the management of hematopoietic cancers such as acute lymphoblastic leukemia and diffuse large B-cell lymphoma, the application of CARs for effectively treating OS metastases remains incompletely defined. Recently, the results of a phase I/II clinical study evaluating the tolerability and activity of HER2-CAR T cells were reported by Ahmed and colleagues. In this study, 16 patients with recurrent and/or metastatic OS were treated with escalating intravenous doses of T cells expressing an HER2-specific CAR with CD28ζ signaling domain (268). Treatment with HER2-CAR T cells was tolerable and induced systemic inflammatory responses represented by elevations of circulating IL-8. Additionally, following infusion persistence of HER2-CAR T cells in circulation could be demonstrate, as well as their trafficking to tumor sites. However, the clinical impact of HER2-CAR T cells was marginal, with three OS patients experiencing stable disease lasting 12-15 weeks, and the remainder of patients having disease progression. While initial results with CAR T cells have yet to meaningfully impact outcomes in metastatic OS patients, strong enthusiasm exists for the continued exploration of additional adoptive cell therapies that include anti-GD2 CAR T cells, natural killer cells, transgenic TCR cells, and γδ T cells.

The development and clinical assessment of adoptive cell therapies have been piloted in canine OS, providing valuable preclinical data regarding feasibility and activity. For CAR T cells, HER2 has also been explored as a target for canine OS, and Mata and colleagues reported the successful development of HER2-CAR T cells that killed HER2+ canine OS cell lines in an antigen dependent manner (269). Additionally, combining radiation and immunotherapy has been recently explored in a first-in-dog trial of autologous natural killer (NK) adoptive cell therapy (270). In this study, OS-bearing dogs were treated with a coarsely fractionated radiation protocol consisting of 9 Gy once weekly for 4 treatments, with NK cells being harvested and expanded *ex vivo*, and then delivered back to dogs by intratumoral injection following the completion of radiation therapy. Of the 10 dogs treated, 5 remained metastasis-free at 6 months, and one had regression of a suspicious pulmonary nodule detected at the time of diagnosis. While preliminary in nature, these studies in canine OS provide the underpinnings to prospectively evaluate different combinatorial strategies inclusive of adoptive cell therapies for combating OS metastatic progression.

## Relevance of Pet Dogs for Realizing Novel OS Metastases Therapeutics

In order for animal models of human pathologies to be useful and informative, it is necessary for the experimental system to be readily available and accurately recapitulate the natural course of disease. For OS, where the genetic underpinnings are chaotic and the etiopathogenesis remains elusive, the creation of experimental model systems becomes fundamentally difficult, and potentially flawed; as the blueprint of models are derived from and constructed upon current understandings of disease processes. Pet dogs that spontaneously develop OS have potential to serve as excellent naturally-occurring models for diverse comparative pathologies, including OS. Through collaborative research efforts, canine OS can be uniquely positioned in the scientific discovery pathway for understanding metastasis biology, which can drive and accelerate the identification of new promising anti-metastatic therapies. Several characteristics of canine OS are noteworthy and with the continued inclusion and future innovative addition of pet dogs as comparative OS models, it would be expected that significant progress will be made toward improving the outcomes of patients diagnosed with OS metastases.

### Abundance, Accelerated Natural Disease Course, and Limited Cure Rate

Pediatric OS, by definition is categorized as a rare tumor (<15 cases per 100,000 people per year). For localized OS, surgery and MAP chemotherapy in pediatric patients produces 5-year event free survival in the majority (60–70%) of patients treated. In this good responder population with favorable histologic Huvos grade, the opportunity to clinically evaluate and realize the anti-metastatic activity of novel therapies is narrow and temporally protracted. While patients with recurrent and/or metastatic OS can be readily included for investigating novel therapies, biologic responses to experimental agents might differ between macro- and microscopic disease settings, and confound the accurate identification of new agents with reproducible anti-metastatic properties.

Canine OS is the most common primary bone tumor in large and giant breed dogs, and has been estimated to affect at least 10,000 pet dogs every year in North America (271), which is a log order greater than the number of pediatric OS patients diagnosed annually in the United States (800–1,000 new cases/year). Conventional treatment with surgery and chemotherapy improves outcomes in affected dogs, producing a median survival time of ~9 months (272). However, even in treated dogs, death as a result of metastatic progression occurs in 85–90% of patients within 2-years of diagnosis. While these statistics for canine OS are sobering, when viewed through the lens of comparative oncology, pet dogs offer an unprecedented opportunity to be included in the evaluation and translation of new anti-metastatic agents. Ultimately, through their purposeful inclusion in drug assessment, pet dogs can accelerate the identification of new agents which hold promise to improve long term outcomes in both humans and canines diagnosed with metastatic OS.

### Comparable Anatomic-Sized Tumor Burdens and Laws of Diffusion

Despite their accepted research value, some limitations of murine models remain irreconcilable including the >10^3^ difference in anatomic size between mice (20 g) and humans (70 kg). Specifically for the evaluation of novel drugs or drug delivery strategies for combating OS metastatic progression, differences in anatomic size and corresponding dimensions of metastatic lesions can strongly bias treatment outcomes simply as a function of tumor volume and interstitial pressures, as therapeutics released into the pulmonary parenchyma are still governed by the physical laws of diffusion prior to reaching their intended targets, being OS metastatic foci. Fick's law of diffusion states that the rate of movement (mass flux) can be modeled mathematically by the following equation:

$$\begin{array}{l} \text{J}=-{\text{D}}^{*}\;\Delta \text{C}/\Delta \text{x} \end{array}$$

**Figure 5:**
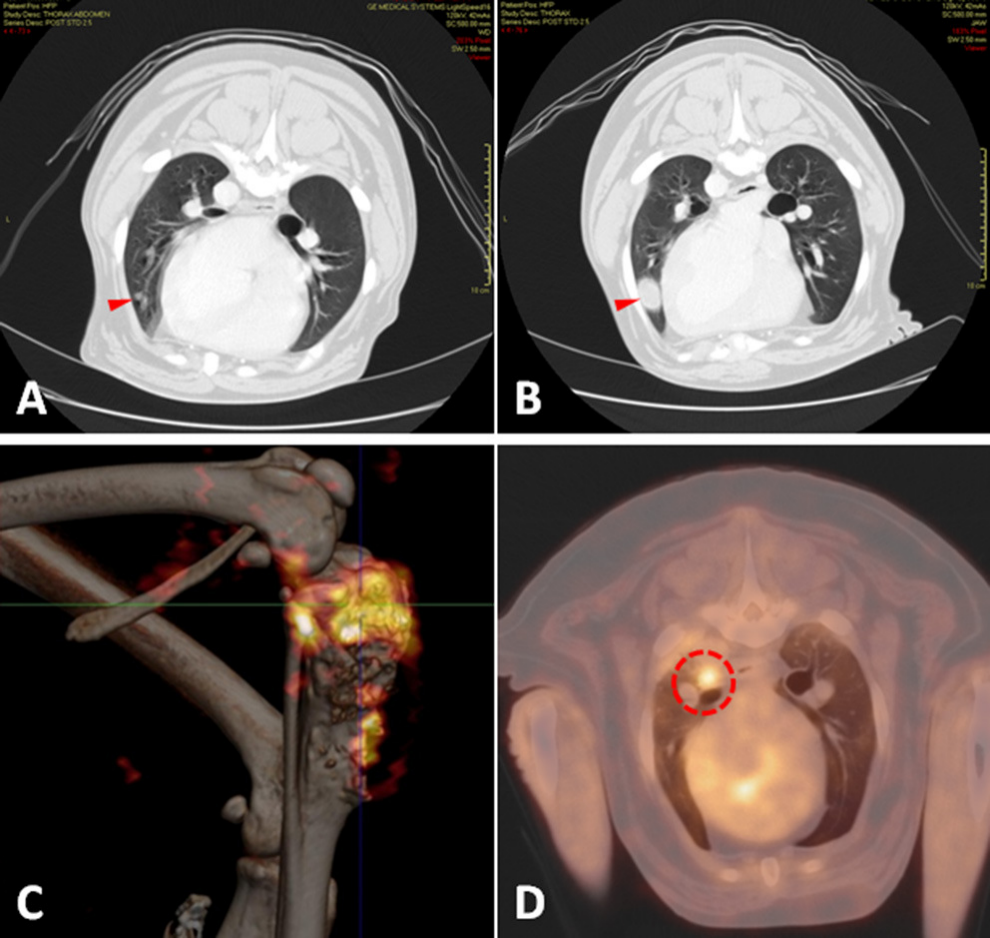
Radiologic assessment and relevance of comparable anatomic size and metabolic activity of OS tumors arising in pet dogs. **(A)** Early detection of emerging pulmonary metastatic lesion (red arrowhead) with **(B)** subsequent rapid macroscopic growth (red arrowhead) over a period of 8 weeks documented by serial CT imaging. Metabolic activity of **(C)** primary bone OS (Image courtesy of Kim Selting, UIUC) and **(D)** pulmonary metastases (Image courtesy of Lynn Griffin, Colorado State University) using PET/CT imaging in pet dogs with OS. UIUC, University of Illinois at Urbana-Champaign.

Where **J** represents mass flux, **D** represents molecular diffusivity of a specific therapeutic agent within a microenvironment, **ΔC** represents the change in concentration gradients, and **Δx** represents distance of diffusion. Although several determinants influence mass flux **J** (representing the movement of therapeutic agents), diffusional distances within a chosen experimental system directly affects the movement rates of therapeutics, thereby having the potential to either positively (small anatomic size and limited diffusion distances) or negatively (large anatomic size and expansive diffusion distances) bias treatment outcomes. As such, drugs that demonstrate potent activity in murine models of OS metastasis can be partially attributed to the diminutive diffusion distances required to be traversed by therapeutic agents. However, when translated to larger mammals including human or canine OS patients, where diffusion distances are log orders greater, achieving therapeutic response becomes more difficult (158), if not impossible as governed by the laws of diffusion. Governed by physical laws of diffusion, studies investigating novel therapies for OS metastases would be most predictive when evaluated in tumor model systems which most closely approximate anatomic sizes at both the organism (human) and target (OS metastases) levels. Under these assumptions, dogs with OS can serve as excellent comparative models for pediatric OS given comparable size in body mass and metastatic tumor burdens (Figures 5A–D) as occurs in both adolescents and giant breed dogs.

### Natural Evolution and Immune Competency

The natural development of cancer is dynamic, giving rise to heterogenous cell populations that in aggregate form solid tumors. Critically contributing to tumor mass evolution are stromal cells within the tumor microenvironment. While specific cell populations like fibroblasts and endothelial cells within the microenvironment can be modeled reasonably well in more sophisticated experimental systems, including mouse models and engineered biomimetics, it remains challenging to recapitulate the dynamic and heterogeneous processes of immune surveillance and editing through existing model systems. With the overwhelming focus on expanding immunotherapeutic strategies for treating various forms of cancer, the inclusion of a model system that firmly mimics immune interactions between cancer and immune effector cells remains of highest priority.

Given the abundance of scientific and clinical evidence supporting OS to be immunogenic in humans and dogs (273, 274), recent investigations have forged new ground which clearly demonstrate the conserved immunologic signatures and phenotypes shared between canine and human OS (275); and these correlative investigations underscore the unique and high valued information that might be gleaned from pet dogs in regards to systemic and immune microenvironment signatures that can be targeted and manipulated to thwart OS progression and metastasis.

A few salient examples of conserved immune targets include *Foxp3* regulatory T cells, tumor-infiltrating macrophages, and tumoral PD-L1 expressions. In dogs with OS, the participation and prognostic value of regulatory T cells in systemic and tumor microenvironmental immunosuppression has been characterized. Biller and colleagues reported that a decreased CD8^+^/T_reg_ ratio was associated with significantly shorter survival times in dogs with OS (276, 277), and these observations in dogs corroborate findings identified in human OS patient samples for the prognostic value of effector/suppressor T cell ratios in predicting long-term outcomes (234). Recently, the significance of tumor-infiltrating macrophages within primary OS lesions have been studied in both dogs and humans. Withers and colleagues characterized the innate and adaptive immune infiltrates within primary OS lesions from 30 dogs and correlated these findings with disease-free interval (278). In this study, the magnitude (dichotomous cutoff of 4.7%) of tumor infiltrating macrophages identified within the primary tumor correlated with survival time, suggesting that macrophages may play an integral role for inhibiting OS metastatic progression. These findings in pet dogs closely mirror some investigations in human OS patients, whereby tumor-infiltrating macrophages were also associated with reduced metastasis and improved survival in patients with high-grade OS (226). Lastly, PD-L1 expressions, which serve as a therapeutic target for checkpoint inhibitor strategies, have been studied in both canine and human OS samples. In canine OS primary samples, Maekawa and colleagues characterized PD-L1 expression across various canine tumor histologies and identified seven out of 10 OS samples to stain positively for PD-L1 (279). Comparatively, the expression of PD-L1 in human OS tissue samples appears more restricted (229), with potential enriched expressions in relapsed or metastatic lesions compared to primary tumors (236, 237). In aggregate, the immune signatures shared between canine and human OS are highly comparable, and provides a rational scientific foundation to leverage pet dogs for evaluating novel immune-based therapies against OS metastases.

## Future Directions

### Research Awareness and Emphasis

Compared to the state of metastasis research in other types of cancer such as breast (~7,703 entries on PubMed) and prostate (~2,653 entries), basic metastasis research in OS (~860 entries) is taking its first furtive steps. Discovering new and critical processes that contribute to OS metastasis biology will pave the way for the development of novel anti-metastatic therapies that can be integrated in the current standard of care. Paradigm shifts in animal research should include a focus on lung colonization, clearing or halting the progression of micrometastases, and shrinkage of established metastases as being translational targets, as recognized and supported by leaders in the OS field (3, 8). Tumor cell dormancy in OS is also an area of much needed research since dormant tumor cells are thought of as a reservoir of future tumor recurrence (280, 281). Funding initiatives that focus on metastasis biology discovery, “omic” approaches in finding actionable targets involved in the metastatic cascade, or re-purposing of existing clinical drugs and assessing their anti-metastatic activity is sorely needed. Such experimental approaches require investment in more animal work, specialized equipment, and skilled personnel. Lastly, shifting the research landscape to include more metastasis-focused initiatives requires a voice from patient advocates, who are an integral part of research funding.

### Necessity and Benefits of Collaborative Science

Fueled by increasing commitments of resources, veterinary oncology collaborative groups have rapidly become more organized, more agile, and more capable of efficiently conducting high-value pilot studies, complex biology studies, and large clinical trials in client-owned pets that develop cancer. The Comparative Oncology Trials Consortium, headquartered at NIH, has undertaken several initiatives to broaden the comparative biology of human and canine cancers and to improve the efficiency with which they can generate pharmacokinetic, pharmacodynamic, and initial efficacy data in canine clinical investigations that can guide human trial development. Recent initiatives arising from the NIH Cancer Moonshot program have recognized opportunities for incorporating canine clinical trials into the evaluation of immune-oncology approaches by funding several large grants and spawning new comparative immuno-oncology consortia.

Intelligent use of the data arising from this work should improve the likelihood of good outcomes in human clinical trials. Coordination across canine clinical consortia, basic scientists, and industry has become increasingly important. The ability to share data and to plan collaboratively has been enhanced by integration of veterinary oncologists into pediatric clinical trials groups and vice-versa. Efforts led by a handful of philanthropic groups to intensify dialog between these stakeholders have met with increasing interest and engagement. These achievements to date represent only initial forays into truly integrated drug development and science—with opportunity far surpassing the current actuality.

### Growth of Comparative Oncology and Coordination of Clinical Trials

With continued and growing interest of the scientific community for including spontaneous tumor models to accelerate novel drug development, comparative oncology centers of excellence must keep pace and commensurately grow to meet the expectations of an ever increasing desire for executing high-value clinical trials in an agile and efficient manner. To achieve these deliverables, existing comparative oncology centers must expand capacity, and new centers must develop. Incentivization for such development can be achieved through different mechanisms including active participation in Clinical and Translational Science Awards (CTSA) program and integration with existing Basic or Comprehensive Cancer Centers.

Central coordination and unification across participating comparative oncology centers should be strongly advocated, thereby increasing the efficiency and impact of ongoing and future prospective clinical trials. Regional consortia operating in silos will not likely maximize collaborative efforts, and have potential to dilute limited shared resources and manpower. Visionary and inclusive leadership in this arena is necessary, and will ensure that all participating teams can be maximally and directionally aligned for the greatest translational impact as possible.

## Conclusions

Cancer metastasis remains the leading cause of mortality for people afflicted with diverse solid tumor histologies. While tremendous advances in therapy have been achieved over the past decade for some tumor types, the management and outcomes of aggressive sarcoma metastases, including OS, remains almost at a standstill. To impact the lives of patients suffering from OS metastases, it will be necessary to deepen our fundamental understanding of OS metastasis and its specific vulnerabilities at both the cellular and microenvironmental levels. Additionally, the translation of new and promising therapeutic discoveries must be evaluated using complementary model systems that faithfully recapitulate natural metastatic disease progression in people. Given the conserved biology of OS in humans and dogs, unique opportunities exist for human and comparative oncology researchers to engage in translationally impactful collaborations, which uniquely include pet dogs with OS to expand the understanding of metastasis biology and clinically realize the activity of novel investigational therapeutics that target OS metastatic progression.

## Ethics Statement

All animal experiments were done with ethics approval of the local Animal Care Committees.

## Author Contributions

TF, RR, and ML: project conception and manuscript authorship.

### Conflict of Interest

The authors declare that the research was conducted in the absence of any commercial or financial relationships that could be construed as a potential conflict of interest.
